# Reveal the Viscoplastic Behaviour and Microstructure Evolution of Stainless Steel 316L

**DOI:** 10.3390/ma15207064

**Published:** 2022-10-11

**Authors:** Qiong Lu, Chi Zhang, Wei Wang, Shuai Jiang, Lee Aucott, Tabassam Yasmeen, Jun Jiang

**Affiliations:** 1 Department of Mechanical Engineering, Imperial College London, Exhibition Road, London SW7 2AZ, UK; 2 Powder Metallurgy Research Institute, Central South University, Changsha 410083, China; 3 United Kingdom Atomic Energy Authority, Culham Centre for Fusion Energy, Culham Science Centre, Abingdon, Oxfordshire OX14 3DB, UK; 4 Advanced Forming Research Centre (AFRC), University of Strathclyde, 85 Inchinnan Drive, Inchinnan PA4 9LJ, UK

**Keywords:** austenitic stainless steel 316L, large grain size, recrystallization, viscoplasticity

## Abstract

Stainless steel 316L is a widely used structural material in the nuclear industry because of its excellent corrosion resistance and mechanical properties. However, very little research can be found on its viscoplastic behaviour and microstructure evolution at warm and hot deformation conditions, which hinder the possible application of advanced manufacturing technologies for producing complex parts, such as superplastic forming or hydroforming. The aims of this study are to explore stainless steel 316L’s viscoplastic behaviour, to determine its strain rate sensitivities, and to reveal its underlying microstructure evolution; this will allow appropriate manufacturing (forming) technologies and the optimal forming condition to be determined. Hence, isothermal tensile tests at 700 °C, 800 °C, 900 °C, and 1000 °C at strain rates of 0.01 s^−1^ and 0.001 s^−1^ have been conducted. Moreover, the corresponding microstructure evolution, including the grain orientation and geometrically necessary dislocation density, has been revealed by the electron backscatter diffraction method. The data show the viscoplastic behaviour of stainless steel 316L under various thermomechanical deformation conditions and how microstructure evolution influences the viscoplastic flow stress.

## 1. Introduction

Using advanced metal forming methods for producing complex-shaped stainless steel 316L (SS316L) parts is highly desirable for future fusion reactor structural components. The SS316L is a member of the austenitic steels that were developed more than three decades ago for fast-breeder reactor applications within EU countries [1,2]. Based on its excellent corrosion resistance, irradiation resistance [3], good thermal conductivity, and low cost [4], SS316L was selected as the main structure retaining material for Vacuum Vessel and the first wall/shielding blanket component (blanket shield block) in the International Thermo-Nuclear Experimental Reactor (ITER) [5,6]. The shielding block has drilled coolant channels which need a multi-layer structure and assemble of different subcomponents to achieve the final complex part [7,8]. This process is challenging and costly, with the assembled larger components under a higher structural integrity risk due to the presence of many long joints [1]. Thus, using advanced manufacturing methods for producing complex-shaped parts with reduced joints is highly desirable for future fusion reactor structural components. Warm or hot forming of complex-shaped components using superplastic forming or hydroforming has increasingly been used in recent years due to its numerous advantages [9,10]. It can produce components with complex geometries, such as high aspect ratio ribbed structures, in one manufacturing step [11]. Therefore, it decreases or completely eliminates the number of sub-components and joining operations [11].

To apply the warm or hot forming techniques in the future fusion reactor structural components, determining the viscoplastic behaviour of SS316L and understanding its underlying microstructure evolution are essential. To achieve the optimal formability of the material, a particular processing window, i.e., specific ranges of deformation temperature and strain rate needs to be identified [12]. The strain rate sensitivity, determined from the ratio of the stress variation to the strain rate difference, is an important formability indicator [13]. For example, a superplastic state with an elongation typically larger than 300% can be achieved if the strain rate value is determined to be ~0.4–0.5 [14]. The lower the value of the strain rate sensitivity, the inferior the formability of the material becomes. This strain rate sensitivity value is governed by the diffusion process-driven microstructure evolution [15,16,17,18]. It was found that the underlying grain size evolution is one of the main intrinsic factors governing the strain rate sensitivity. Moreover, the grain size plays a vital role in determining the fracture toughness, fatigue, creep, and corrosion resistance of the formed components [19]. Hence, it is important to explore the relationship between grain size and various thermal-mechanical conditions [20,21]. Such a relationship has been extensively studied among AISI 304L stainless steel [22] and super-304H austenitic stainless [23], as well as 410 stainless alloys [24]. However, so far, little research has been devoted to investigating the viscoplastic behaviour of SS316L, which is one of the most used structural materials for fusion reactors.

The aim of this study is to reveal the viscoplastic behaviour and underlying microstructure evolution of SS316L. To obtain the stress–strain curves, eight samples will be hot deformed at various testing temperatures and strain rates: 700 °C, 800 °C, 900 °C, and 1000 °C, at strain rates of 0.01 s^−1^ and 0.001 s^−1^, respectively. Based on these obtained stress–strain curves, the strain rate sensitivity calculation will be undertaken as a function of deformation temperature and strain. Moreover, the underlying grain size, grain orientation, and geometrically necessary dislocation (GND) density will be analysed by the electron backscatter diffraction (EBSD) technique to provide insights into microstructure evolution.

## 2. Experimental Methodology

### 2.1. As-Received Material and the Testing Samples

**Figure 1:**
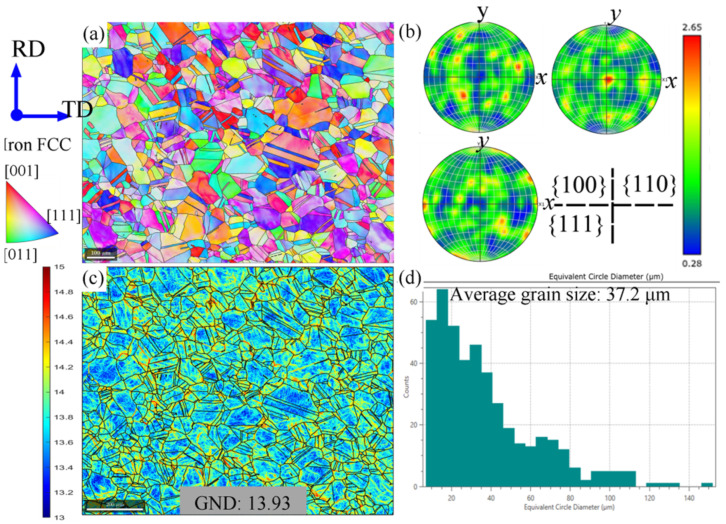
Microstructure of as-received SS316L: (**a**) EBSD IPF images, (**b**) pole figures, (**c**) GND density map, (**d**) grain size distribution.

The as-received sample, its preparation for testing, and detailed testing procedures are described here. ASTM A240 316L steel rods with a diameter of 20 mm were provided by Masteel, Lichfield, UK. Its chemical composition is reported in Table 1 and microstructure was characterized using EBSD, as seen in Figure 1. Figure 1a,b depicts the grain orientation map by the inverse pole figure (IPF) and preferential orientation distribution, i.e., texture, through the pole figure (PF). The initial microstructure consists of equiaxed grains with little noticeable texture. Moreover, it confirms the austenitic phase (iron FCC) structure. In these FCC austenitic grains, moderate GND content (the average GND value of the bulk is 13.93 on the log_10_ scale) can be found, as shown in Figure 1c. The presence of these moderate GND density points implies that the initial material is likely in a wrought state without heat treatment. Based on a statistical analysis, the grain size is relatively uniform with a moderate average of ~37 μm, as shown in Figure 1d. Moreover, a large fraction of annealing twins exist in the microstructure due to the low stacking fault energy of the 316L [25,26]. These twin boundaries were identified according to the specific misorientation (60°) with the axis (<111>). The quantitative data of the twinned area and twinned grain fraction were calculated and are shown in Table 2. Note that these twin boundaries were excluded from the previous grain size analysis in Figure 1d.

**Figure 2:**
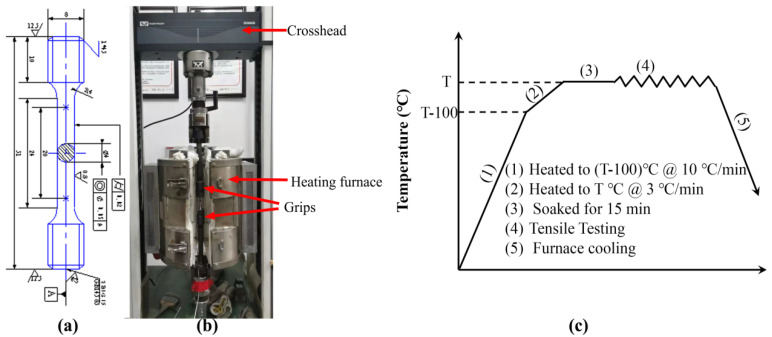
(**a**) Dimensions of the uniaxial hot tensile specimen, the unit in this figure is mm (**b**) hot tensile testing system used in this study, and (**c**) schematic representation of the thermo-mechanical processing route.

Using a wire-cut electrical discharge machine, these rods were machined into uniaxial hot tensile testing samples, according to the ASTM E-2448 standard [28], as presented in Figure 2a. These machined samples were subjected to the hot tensile test using the Instron 3369 thermal-mechanical testing machine (Instron, Boston, MA, USA), as shown in Figure 2b. It can operate from room temperature to 1200 °C with ±2 °C accuracy using an attached two-halves-split furnace. The pull rods of the machine are equipped with cylindrical bearings at both bar ends: upper and lower. This set-up ensures good alignment between the specimen axis and the loading axis. The pull rods were joined to specimen grip boxes using short, double-threaded link rods. The displacement was measured by an extensometer (CBY-DG 25-5), attached to the gauge region of the specimens.

The testing program is schematically illustrated in Figure 2c, with the testing sample first heated to 100 °C lower than the designed testing temperature with a heating rate of 10 °C per minute; the heating rate was then reduced to 3 °C per minute to heat the sample to the designed temperature that would minimize the temperature overshooting issue. To ensure temperature uniformity along the sample, the sample was soaked at the designed temperature for 15 min prior to the onset of loading. The loading process was undertaken using displacement control, according to the designed strain rate, until the occurrence of the fracture; then, the sample was furnace cooled to the room temperature. Uniaxial hot tensile experiments were conducted at two strain rates of 0.01 mm/s and 0.001 mm/s, and four temperatures of 700 °C, 800 °C, 900 °C, and 1000 °C, respectively.

During the hot tensile tests, argon was used as a protective gas to minimize the surface oxidation issue. Software modification and varying crosshead speeds were calibrated to obtain a constant strain rate. The optimum temperature, strain rate, and strain rate sensitivity index values were determined. Compared to conventional hot tensile testing, this ultra-high temperature, ultra-low strain rate test takes a significantly longer time and is difficult to perform; thus, only one test per test condition was conducted in this study.

### 2.2. EBSD Characterization

**Figure 3:**
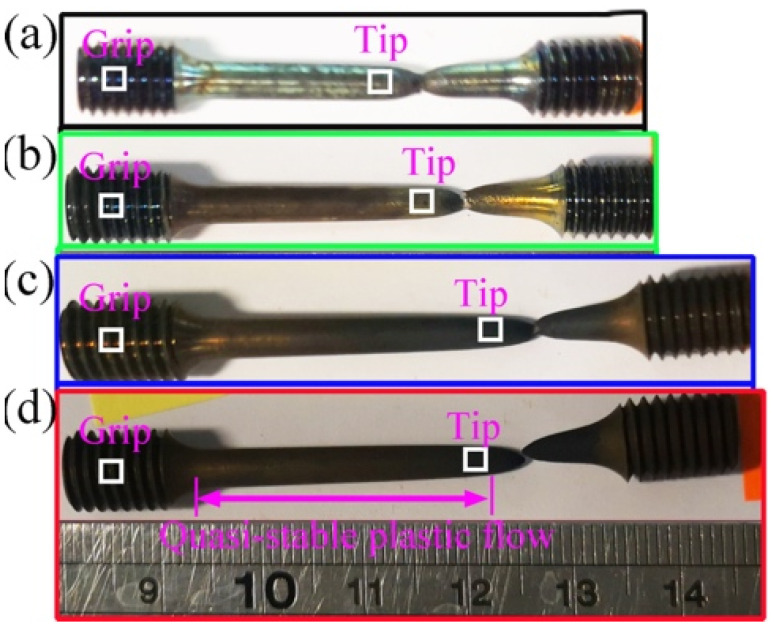
Macroscopic morphology of specimens after tensile test: (**a**) 700 °C, (**b**) 800 °C, (**c**) 900 °C, and (**d**) 1000 °C at a strain rate of 0.01 s^−1^. The highlighted white squares are the selected EBSD regions.

EBSD characterization was conducted on the as-received and deformed samples. The EBSD samples were cut from the grip and gauge regions, as displayed in Figure 3, to isolate the heat treatment effect from viscoplastic deformation. The EBSD samples were then ground down using SiC paper, progressively from 600 to 4000 grits. Subsequently, the ground samples were polished with 1 μm diamond paste and oxide polishing suspension (OPS) for 45 min, respectively. These metallurgical prepared samples were placed in a SEM (Tescan Clara, Brno, Czech Republic), in which 20 keV acceleration voltage and 10 nA current were set. Oxford Instrument EBSD system Symmetry 2 was used to acquire relatively large EBSD maps, i.e., 1400 μm × 1050 μm, with a step size of 2 μm at 150× magnification. The selected map size and step size were due to the balance of the statistically meaningful sampling and containing distinguishable dislocation information (dislocation channels). A smaller area of 600 μm × 435 μm with a finer step size of 0.8 μm was obtained for closer inspection. The EBSD pattern indexing rate exceeds 95%; hence, there is little background noise and no artificial points were added to our quantitative analyses. The step size of 0.8 μm was selected based on the grain size and its distribution. As the grain size here is relatively large, 0.8 μm is sufficient to provide meaningful statistical analysis.

As we know, dislocations present in crystal lattices can be subdivided into geometrically necessary dislocations (GNDs) and statistically stored dislocations (SSDs), depending on their overall contribution to lattice curvature [29]. Compared to several dislocation characterization techniques, such as transmission electron microscopy (TEM) [30,31], X-ray diffraction (XRD) [32], neutron diffraction (ND) [33], and chemical pitting [34], the EBSD technique is an inter bridge that fills the length-scale gap between the TEM and X-ray diffraction, which allows the observation of the dislocation structures and in the meantime, gives a quantitative average dislocation density value over a relatively large mapping area [35]. Thus, we use this EBSD method to characterize the GND distribution. The grain size and GND density were analysed using the MTEX toolbox, with the grain boundary set as 10° misorientation.

## 3. Results and Discussion

### 3.1. Stress–Strain Behaviour

**Figure 4:**
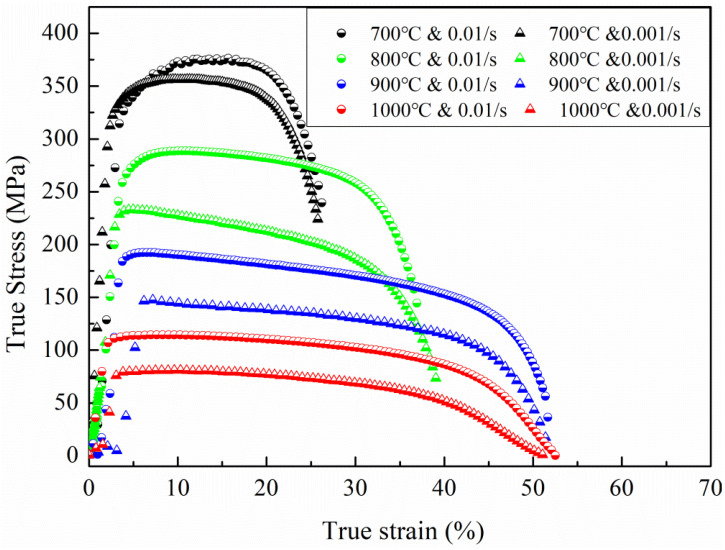
The effect of temperature and constant strain rates on stress–strain behaviour.

The true stress–strain curves at various temperatures and strain rates are reported in Figure 4. It can be seen that the flow stress is very sensitive to the change in deformation temperature and strain rates. All curves exhibit peak stress rapidly after the initial hardening. These peak stresses decreased gradually with increasing temperature and decreasing strain rate. At the high strain rate, the peak stress decreased from 361 MPa to 111 MPa as the temperature increased from 700 °C to 1000 °C. At 700 °C, the curve displays a steady state after a peak stress, which means that the dynamic softening and hardening reached an equilibrium. As the deformation temperature increases from 800 to 1000 °C, the curves exhibit a continuous flow softening regime after the peak upward stress, which suggests the predominant softening effects exerted by dislocation recovery, recrystallization, or grain boundary sliding [36,37,38].

The elongation of the specimens generally increases with higher temperatures, while the strain rate seems to have little influence on the ductility. The highest elongation of 50% is observed at 1000 °C, whereas the lowest elongation (~20%) is found at the lowest testing temperature of 700 °C.

The strain rate sensitivity index, *m*, which is generally accepted as a formability indicator, was calculated using the following equation [39]:

$$m=\frac{\partial \ln\sigma }{\partial \ln\dot{\varepsilon }}|T,\varepsilon$$

where *σ* is the flow stress, $\dot{\varepsilon }$ is the strain rate, *T* is the absolute temperature in kelvin, and *ε* is the true strain.

**Figure 5:**
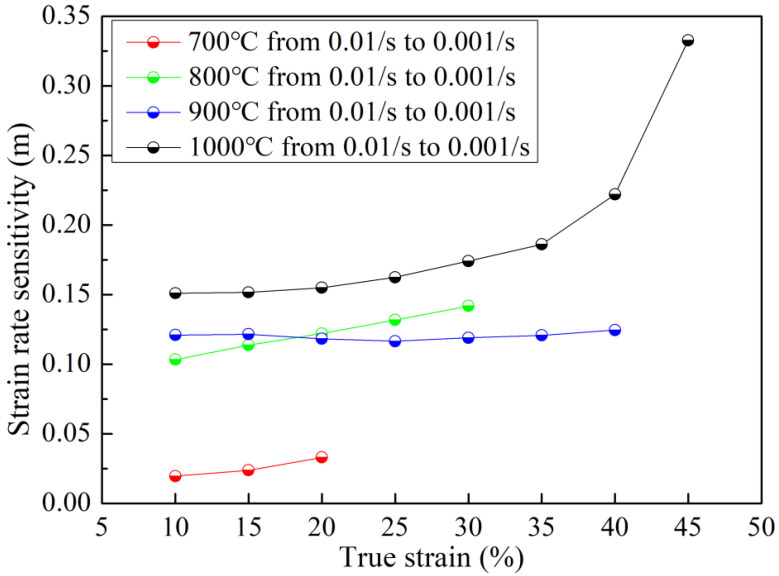
Strain rate sensitivity evolution with deformation temperature.

An *m* value of 0.33 was achieved at a strain of 45% at 1000 °C, which is approximately twice higher than that at its 20% strain. The results are reported in Figure 5. The *m* value increases gradually with increasing temperature. For example, *m* is determined as 0.03, 0.12, 0.12, and 0.15 at 700 °C, 800 °C, 900 °C, and 1000 °C, respectively. Low *m* values (less than 0.1) indicate low strain rate sensitivity, and hence have the tendency to strain localization and necking, which means poor formability. However, at 1000 °C, there is an increasing trend for the strain rate sensitivity, which increased from 0.15 at a strain level of 20% to 0.33 at a strain level of 45%. An *m* value of 0.33 is very high, which means that if the microstructure at this state could be maintained as a steady state during the deformation, the elongation of the materials could be very large (>300%), since *m* = 0.33 is defined as a superplastic condition in the literature [36,40]. In a physical sense, it means that the microstructure at this strain level has sufficient diffusion taking place at the grain boundaries, which allows grain boundary sliding, the predominant mechanism for superplasticity, to occur [41].

### 3.2. Grain Evolution

**Figure 6:**
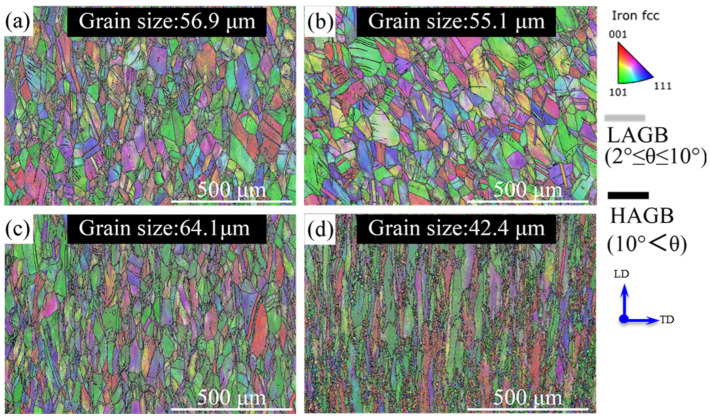
Grain orientation maps of SS316L deformed at 0.01 s^−1^ strain rate and different tensile temperatures of: (**a**) 700 °C, (**b**) 800 °C, (**c**) 900 °C, and (**d**) 1000 °C, respectively.

EBSD orientation maps under various deformation temperatures with 0.01 s^−^^1^ strain rate exhibit considerable grain size changes from 700 °C to 1000 °C, as shown in Figure 6. Both the high angle grain boundaries and the low angle grain boundaries (LAGB) are highlighted in the maps. The grains are elongated along the vertical axis, which corresponds to the loading axis. The grain sizes of all four conditions are found to be larger than that of the as-received grains. Moreover, more LAGB exist in the higher temperature deformed samples. Initially, the grains size increased gradually with increasing tensile temperature and reached a maximum of 64.1 μm at 900 °C, which is ~72% larger than that of the as-received alloy. This is the main reason for the decrease of the *m* value with the strain increasing under deformed conditions at 900 °C, as the grain size is one of the main influencing factors of the *m* value [15]. In contrast, when the deformation temperature increases to 1000 °C, it is interesting to note the significant decrease in grain size (42.4 μm, which is ~34% smaller than that at 900 °C), due to the occurrence of dynamic recrystallization (DRX). The recrystallized grains can be distinguished by their smaller size (Figure 6d) and their equiaxed shape. Clear necklace microstructures emerged near grain boundaries.

The recrystallization was further analysed using the method of grain orientation spread (GOS) within grains [42]. Identifying the recrystallized grains is challenging, since the recrystallized grains are deformed simultaneously once they are generated. It is often hard to distinguish them from the original grains. Nevertheless, in this study, the recrystallized grains are in their early stage and their size tends to be significantly smaller than the original grains. Moreover, the recrystallized grains could be formed during the cooling process, etc., post deformation recrystallization. Thus, their orientation spread within grains is low. Therefore, it is reasonable to utilize the GOS to distinguish the recrystallized grains.

**Figure 7:**
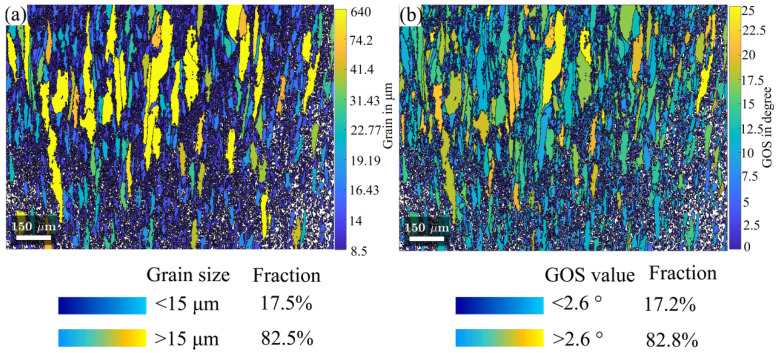
Identification of recrystallization grains of SS316L deformed at 1000 °C with 0.01 s^−1^ strain rate is based on grain orientation spread: (**a**) distribution of grain size, (**b**) distribution of grain orientation spread.

In this study, the grains with a GOS value below 2.6° were identified as dynamic recrystallized (DRXed) grains, as referred to in [43]. The grain size and GOS criteria for distinguishing recrystallized grains have been compared, as shown in Figure 7. The new DRXed grains with equiaxed shapes presented a homogeneous grain size (~15 μm) distribution and the DRX fraction is approximately 17.5% (as shown in Figure 7a), which is consistent with the cumulative area fraction at GOS smaller than 2.6° (as shown in Figure 7b). These indicate that the 2.6° threshold used by GOS to distinguish DRXed is sensible.

In this sample, LAGBs filled the unDRXed region and the unDRXed grains exhibited a relatively large size and were dramatically elongated (Figure 6d). These LAGBs are formed by the pile-up of dislocations with the same sign. The high content of LAGB indicates the high dislocation density, etc., plastic strain energy stored in the material. As DRXed grains form near the grain boundaries with a necklace structure of equiaxed grains form, with the SS316L having relatively low stacking fault energy, which means its dislocation recovery rate is low. Thus, the current DRX is discontinuous DRX, which is consistent with previous DRX studies in SS316L [44]. In addition, it can be seen from Figure 7 that grain size had a smooth gradient distribution, decreasing gradually from the top part to the bottom part. The fraction of DRX increased with an increasing deformation degree [45]. 

**Figure 8:**
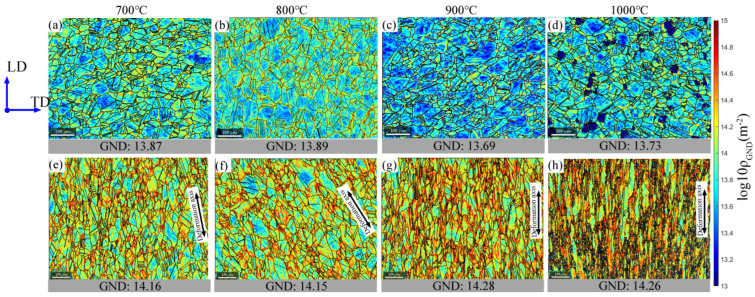
GND maps of SS316L deformed at 0.01 s^−1^ strain rate and different tensile temperatures. Note that (**a**–**d**) are grip areas and (**e**–**h**) are tip areas. The colour bar shows GND density on the log_10_ scale of line m^−2^.

As dislocation density is the main driving force for discontinuous DRX [46], to analyse the dislocation change, the GND density, which results in lattice geometrical change and curvature by the presence of dislocations with the same sign, was obtained by measuring the local orientation change from EBSD [47], with the results shown in Figure 8.

### 3.3. GND Density Evolution

The effects of the static annealing process under various testing temperatures are revealed by checking the GND densities in the grip parts of the sample, which is subjected to static annealing without involving any deformation, and are presented in Figure 8a–d. It can be inferred that, since the as-received material has a large dislocation content, the overall GND density decreases gradually with the increasing annealing temperature, suggesting a more active recovery process as the temperature and diffusion process increase. At 1000 °C, a few small grains are dislocation-free, as shown in the dark blue regions in Figure 8d, which indicates that static recrystallization has to be activated through the increasing thermal energy [15,48].

It is interesting to see the increase of GND density in the deformed region as a function of increasing deformation temperature. The previous study on hot compression 316L to 50% at various temperatures showed a strong positive correlation between the flow stress and the GND density [49]. However, this trend does not show in the current study. If we consider the various plastic strain levels in the current study, it is less surprising that the GND is measured at the end of the test, for which the materials exhibit higher elongation with increasing temperatures. Although there is no significant hardening, GND density tends to increase with plastic strain. It should be noted that EBSD maps were captured near the fracture tip where the plastic strain and strain rates could be much higher than the nominal ones. In general, at the provided deformation temperature, the average stored GND contents increased with the increase in plastic deformation [50]. Considering the specimen that deformed at 700 °C, the averaged GND density increased from 13.9 to 14.16 on the log_10_ scale (as shown in Figure 8a,e), as the strain increased from 0 to 20%. This trend is consistent at 800 °C, 900 °C, and 1000 °C by comparing the grip and deformed regions.

A closer examination of the GND structures in Figure 8e–h reveals that the GNDs gather near the grain boundaries, especially around the triple junctions, which is consistent with the GND structure formed under room temperature deformation [51], while most of the high-temperature-induced GNDs also tend to form channel-like structures across the bulk matrix. These channel-structured GNDs could be identified from the accumulated red and yellow colours inside the grains. The channels are often parallel with the direction of grain elongation, which is the same with the deformed axis. According to the colour difference, we can identify that the GND structure progressively expands along the loading axis and the river-like pattern starts to develop.

### 3.4. Dynamic Recrystallization and Twin Boundaries

**Figure 9:**
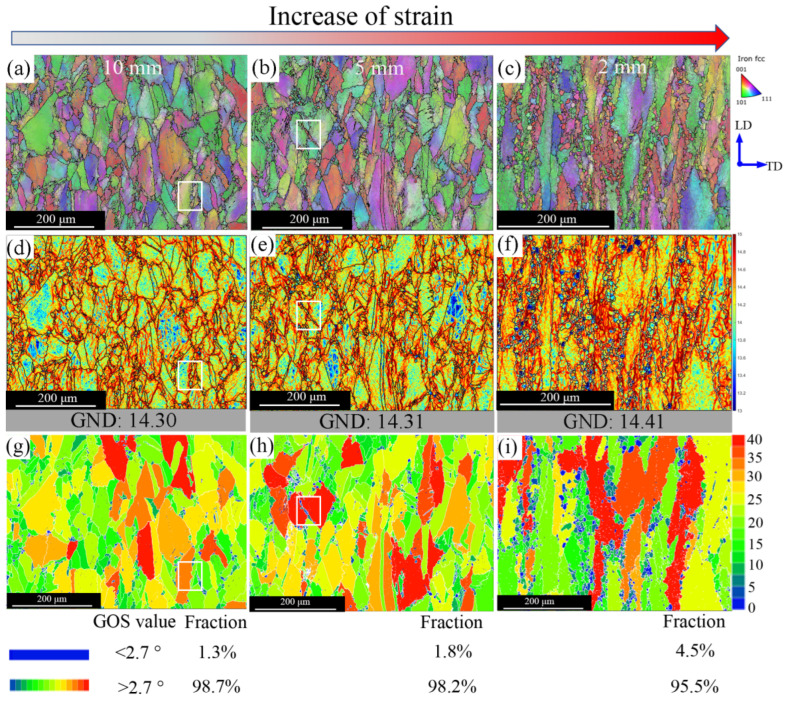
(**a**–**c**) EBSD IPF maps of different positions deformed at 1000 °C with 0.01 s^−1^ and (**d**–**i**) their corresponding GND density and GOS maps, respectively.

To provide more insight into the underlying nucleation mechanisms and discover why most new grains nucleate at grain boundaries, higher spatial resolution EBSD maps were made at the different stages of dynamic recrystallization. As the driving force of the recrystallization is the dislocation density [52], regions with different dislocation density, as shown in Figure 9a–c, are selected from the gauge part due to the non-uniform deformation. For these three regions, the distances from the fracture tip area are 10 μm, 5 μm, and 2 μm, respectively. GND density and GOS analyses are undertaken, with the results presented in Figure 9d–i. 

**Figure 10:**
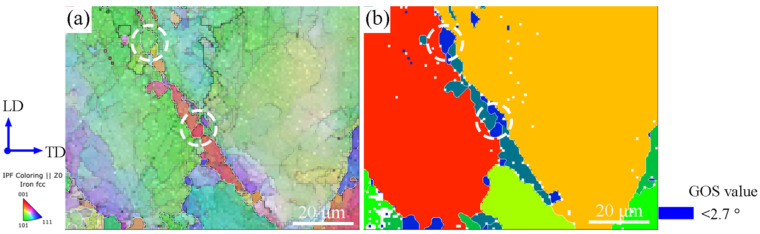
Grain nucleation at twin boundaries of the deformed grain from the boxed area in Figure 9b: (**a**) IPF image, (**b**) GOS image.

The recrystallized fraction increases with increasing strain. Some of the regions are highlighted (marked in white squares) in Figure 9a,d,g. It can be seen that at the beginning of recrystallization, grain boundaries become serrated due to the dislocation density gradients close to the boundaries, with the grain boundary serration occasionally accompanied by local sub-boundaries formation. With the strain increasing, the deformation twins are formed (as shown in Figure 9b,e,h), which also seem to play an important role in the nucleation mechanism. Twinning induces a change in the boundary misorientation and possibly higher mobility, eventually leading to grain nucleation [53,54]. As can be seen on the GOS map (Figure 10, the magnification of the white box in Figure 9b), the new recrystallized grains, having a low dislocation density (blue colour corresponding to a low GOS value, as shown in Figure 10b), are only separated by a twin boundary from the deformed grain. Therefore, it can be concluded that deformation twin boundaries seem a preferential nucleation site for recrystallization happening.

### 3.5. Viscoplastic Mechanism

**Figure 11:**
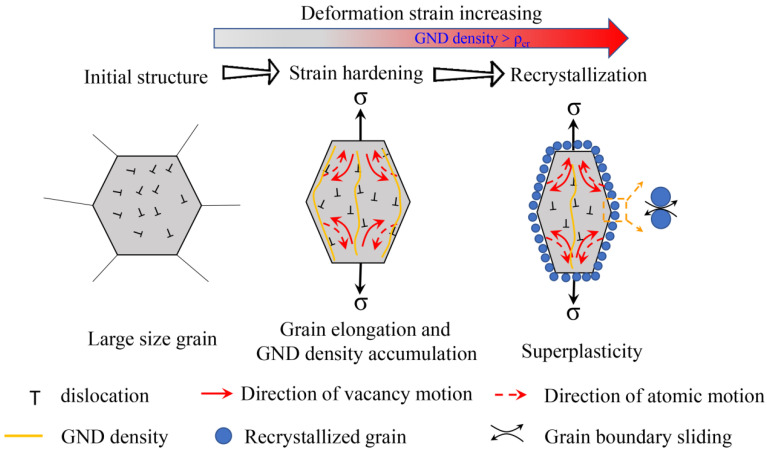
Schematic illustration of the deformation mechanism evolution during high temperature tensile test.

A schematic illustration of the deformation mechanism evolution during the high temperature tensile test is shown in Figure 11. At 900 °C, the strain rate sensitivity value is ~0.125, which indicates that the deformation mechanism is under diffusional creep [55]. It is speculated that the underlying mechanism is driven by vacancy movement along the grain boundaries. The grain boundary perpendicular to the external loading axis is stretched and the grain boundary parallel to the external force axis is compressed. Because the grain boundary itself is the source and annihilation well of the vacancies [56], the formation energy of the vacancies perpendicular to the force axis is low and the number of vacancies is large. However, the grain boundary vacancies parallel to the force axis have higher vacancy formation energy and fewer vacancies, resulting in the formation of certain vacancy concentration difference vacancies in the grain interior flowing in the direction of solid arrows and atoms flowing in the direction of dotted arrows, resulting in plastic deformation with elongation. As the vacancy aggregates form a dislocation, a channel-like structured GND will be formed within the elongated grains during the high temperature tensile test. At the same time, this deformation mechanism limited the plasticity of the matrix which is consistent with the stress–strain curve and strain rate sensitivity value, as shown in Figure 4 and Figure 5, respectively.

Increasing the tensile deformation temperature to 1000 °C results in both reduced flow stress and an increased *m* value at strain rates of 0.01 s^−1^ and 0.001 s^−1^, with the corresponding ductility rising from approximately 26% (700 °C with a strain rate of 0.01 s^−1^) to 53% (1000 °C with a strain rate of 0.01 s^−1^). One advantage of recrystallization is its potential ability to sustain an ultrafine-grained microstructure. The resulting grain refinement then facilitates the sliding of grains, which reduces the effective stress. Besides affecting size, the globular microstructure is beneficial to boundary sliding and grain rotation during superplastic deformation because the elongated morphology is not favourable for the interphase boundary sliding. Thus, the recrystallization during deformation plays a critical role in superplastic deformation.

## 4. Conclusions

The viscoplastic deformation behaviour of SS316L has been revealed under the testing temperature range from 700 °C to 1000 °C and strain rates of 0.01 s^−1^ to 0.001 s^−1^. Meanwhile, the underlying microstructure evolution, including grain size and GND density, has been presented. The following main conclusions can be drawn:

(1) The GND density and grain size evolution during viscoplastic deformation (700–1000 °C with strain rates of 0.01 s^−1^ to 0.001 s^−1^) of 316L stainless steel (single phase FCC iron) has been revealed by the EBSD technique. In this study, the estimated GND density seems representative of total dislocation density, successfully rationalising the classic viscoplastic hardening and softening behaviour, such that EBSD-based dislocation density measurement can potentially be used for directly validating physically based viscoplastic constitutive models.
(2) The strain rate sensitivity of 316L exhibits an interesting trend, in that it is generally low (0.1–0.2) at 700–900 °C but increases to 0.33, at 45% strain at 1000 °C. This high strain rate sensitivity is found due to the occurrence of DDRX. These findings suggest that the careful control of DRX could enable the materials with initially coarse grains structure to enter their superplasticity states.
(3) Further investigation revealed this high strain rate sensitivity and significant DDRX are stimulated and promoted by the formation of deformation twins. Their boundaries seemed to be very effective to pin dislocations and subsequently became grain nucleation sites, when compared to other random high angle grain boundaries.
(4) The EBSD-estimated GND density distribution provided evidence of the diffusion creep of large-sized grains. The vacancy-driven deformation mechanism for 900 °C and 1000 °C viscoplastic behaviour was confirmed. These again demonstrate the powerful EBSD-based dislocation density measurement approach, enabling both quantitative and detailed in-depth qualitative analyses for materials under viscoplastic deformation.

## Figures and Tables

**Table 1:** Nominal chemical composition of SS316L [27].

Element	Fe	C	Mn	Si	Cr	Ni	Mo
Wt.%	Bal	<0.03	1.5	0.5	16–18	10–14	2–3

**Table 2:** The details of twinning.

Twinned Grains Count	Twinned Grains Fraction (%)	Twinned Grain Area Fraction (%)
247	55.88	87.99

## Data Availability

This article has no additional data. All experimental and numerical results are reproducible.
